# Supplementary material for: The experience of trial participation disclosure among sex workers in a phase IIb HIV vaccine trial: A qualitative study in urban Tanzania
Source: PLOS Glob Public Health. 2025 Nov 19;5(11):e0005511. doi: 10.1371/journal.pgph.0005511 (PMC12629454; doi:10.1371/journal.pgph.0005511)
Supplement: S1 File — (PDF) [file pgph.0005511.s001.pdf]

COREQ checklist

The Experience of Trial Participation Disclosure Among Sex Workers in a Phase IIb HIV Vaccine Trial: A Qualitative Study in Urban Tanzania

Item No	Guide Questions/Description	Comment	Reported on Page # or N/A
**Domain 1: Research team and reflexivity**			
**Personal Characteristics**			
1. Interviewer/ facilitator	Which author/s conducted the interview or focus group?	The lead author JSA and GL conducted the interviews	6
2. Credentials	What were the researcher's credentials? E.g., PhD, MD	Authors' credentials range from professors to PhD and master's degrees holders. All authors trained in qualitative research and have extensive experience in conducting qualitative health research and completed study training.	N/A
3. Occupation	What was their occupation at the time of the study?	Authors were either university professors, lecturers, assistant lecturers, or researchers at a university or research institute	1
4. Gender	Was the researcher male or female?	Six researchers were males and four females	1
5. Experience and training	What experience or training did the researcher have?	All research team members have training in conducting qualitative health research.	N/A
**Relationship with participants**			
6. Relationship established	Was a relationship established prior to study commencement?	Interviewers (JSA and GL) did not have any relationship with participants in this study to ensure that the collection of data was unbiased.	N/A
7. Participant knowledge of the interviewer	What did the participants know about the researcher? e.g. personal goals, reasons for doing the research?	Participants were introduced to the researchers at the beginning of each interview. Participants were informed about the study's purpose and procedures by the researchers	8
8. Interviewer characteristics	What characteristics were reported about the interviewer/facilitator? e.g. Bias, assumptions, reasons and interests in the research topic	Interviewers were bilingual i.e. English and Kiswahili speaking	N/A
**Domain 2: study design**			
**Theoretical framework**			
9. Methodological orientation and Theory	What methodological orientation was stated to underpin the study? e.g. grounded theory, discourse analysis, ethnography, phenomenology, content analysis		4
**Participant selection**			
10. Sampling	How were participants selected? e.g., purposive, convenience, consecutive, snowball		5
11. Method of approach	How were participants approached? e.g., face-to-face, telephone, mail, email		6
12. Sample size	How many participants were in the study?		8
13. Non-participation Setting	How many people refused to participate or dropped out? Reasons?		5

Item No	Guide Questions/Description	Comment	Reported on Page # or N/A
14. Setting of data collection	Where was the data collected? e.g., home, clinic, workplace		6
15. Presence of nonparticipants	Was anyone else present besides the participants and researchers?	No one else was present besides the participants and researchers	N/A
16. Description of sample	What are the important characteristics of the sample? e.g. demographic data, date		8
	**Data collection**		No
17. Interview guide	Were questions, prompts, and guides provided by the authors? Was it pilot tested?		6
18. Repeat interviews	Were repeat interviews carried out? If yes, how many?	The participants were interviewed at three-time points during the trial – months 2, 6, and 12 as part of the study data collection plan	6
19. Audio/visual recording	Did the research use audio or visual recording to collect the data?		6
20. Field notes	Were field notes made during and/or after the interview or focus group?		6
21. Duration	What was the duration of the interviews or focus group?		6
22. Data saturation	Was data saturation discussed?		6
23. Transcripts returned	Were transcripts returned to participants for comment and/or correction?	Transcripts were not returned to participants for comment and/or correction but member checking was done during the IDIs and FGDs	N/A
	**Domain 3: analysis and findings**		
	**Data analysis**		
24. Number of data coders	How many data coders coded the data?		7
25. Description of the coding tree	Did the authors provide a description of the coding tree?		7
26. Derivation of themes	Were themes identified in advance or derived from the data?		7,8
27. Software	What software, if applicable, was used to manage the data?	No software was used	N/A
28. Participant checking	Did participants provide feedback on the findings?	Feedback on findings was not provided to participants	N/A
	**Reporting**		
29. Quotations presented	Were participant quotations presented to illustrate the themes/findings? Was each quotation identified? e.g., participant number		9-21
30. Data and findings consistent	Was there consistency between the data presented and the findings?		9-21
31. Clarity of major themes	Were major themes clearly presented in the findings?		8-21
32. Clarity of minor themes	Is there a description of diverse cases or a discussion of minor themes?		9-21
