# Supplementary material for: The experience of trial participation disclosure among sex workers in a phase IIb HIV vaccine trial: A qualitative study in urban Tanzania
Source: PLOS Glob Public Health. 2025 Nov 19;5(11):e0005511. doi: 10.1371/journal.pgph.0005511 (PMC12629454; doi:10.1371/journal.pgph.0005511)
Supplement: Attachment — Submitted filename: Response to Reviewers.docx [file pgph.0005511.s003.docx]

**The Experience of Trial Participation Disclosure Among Sex Workers in a Phase IIb HIV Vaccine Trial: A Qualitative Study in Urban Tanzania Response to reviewer’s comments**

We would like to thank the reviewer for the comments provided to improve this study. We have revised the manuscript as shown in the point-to-point responses to reviewer’s comments provided in the table below.

| **Comments** | **Authors’ Response** |
| --- | --- |
| **Journal requirements:** |  |
| 1. Please ensure you have included the registration number for the clinical trial referenced in the manuscript. | Thank you for your comment. We have now included the clinical trial registration number (NCT04066881) in the manuscript, inserted immediately after the abstract. |
| 2. Your current Financial Disclosure states, “This study was funded by The Second European & Developing Countries Clinical Trials Partnership (EDCTP2); (Grant reference: RIA2016 -1644). An additional funding support was also received from Gilead (CO-UK-412-5430).”. However, your funding information on the submission form indicates that you received funding from “Center for Clinical Trials, Japan Medical Association”. Please indicate by return email the full and correct funding information for your study and confirm the order in which funding contributions should appear. Please be sure to indicate whether the funders played any role in the study design, data collection and analysis, decision to publish, or preparation of the manuscript. | Thank you for noting this discrepancy. This study was funded by the Second European & Developing Countries Clinical Trials Partnership (EDCTP2); (Grant reference: RIA2016 -1644). An additional funding support was also received from Gilead (CO-UK-412-5430). The funders did not play any role in the study design, data collection and analysis, decision to publish, or preparation of the manuscript.  Please omit the information on the submission form that this study was funded by the “Center for Clinical Trials, Japan Medical Association”. This is incorrect and was an oversight.  We have also written a return email the full and correct funding information for our study and the order in which funding contributions should appear. |
| 3. Your manuscript is missing the following sections: Result. Please ensure these are present, and in the correct order, and that any references to subheadings in your main text are correct. An outline of the required sections can be consulted in our submission guidelines here:   https://journals.plos.org/globalpublichealth/s/submission-guidelines#loc-parts-of-a-submission | Thank you for the comment. Our manuscript used the term ‘Findings’ for the results section which is more acceptable and relevant to qualitative studies. However, we have made revision and used the term ‘Results’ to comply with the journal’s requirements. |
| **Reviewer #1** | Authors’ Response |
| 1. This is an excellent paper on the reasons for disclosing/not disclosing HIV vaccine participation among sex workers, and I feel that it may be a valuable contribution to the body of literature on stigma surrounding HIV and its treatment, especially among special populations such as the one focused on in this study.   I was especially impressed by the readability of the data shared - there are a variety of testimonies included, and the categorization of the different reasons and flow of the narrative was easy to read. The conclusions were sound, and the implications for further research into HIV vaccines was thought-provoking.  My sole minimal concern is that it may be beneficial to include a description of the randomization process, especially as the sample size is relatively small  All in all, the submitted manuscript reads very well, and I have no major concerns about the quality of the paper. | We thank the reviewer for the positive feedback, noting that this is an excellent paper with potential to contribute meaningfully to the literature on HIV-related stigma and treatment. In response to the comment, we have revised the sampling subsection in the Methods section and added details on the randomization process used to select the 15 volunteers. |
