# Supplementary material for: The experience of trial participation disclosure among sex workers in a phase IIb HIV vaccine trial: A qualitative study in urban Tanzania
Source: PLOS Glob Public Health. 2025 Nov 19;5(11):e0005511. doi: 10.1371/journal.pgph.0005511 (PMC12629454; doi:10.1371/journal.pgph.0005511)
Supplement: Attachment — Submitted filename: Response to Reviewers_2.docx [file pgph.0005511.s004.docx]

**The Experience of Trial Participation Disclosure Among Sex Workers in a Phase IIb HIV Vaccine Trial: A Qualitative Study in Urban Tanzania**

**Response to reviewer’s comments**

We would like to thank the reviewer for the comments provided to improve this study. We have revised the manuscript as shown in the point-to-point responses to reviewer’s comments provided in the table below.

| **Comments** | **Authors’ Response** |
| --- | --- |
| **Journal Requirements:** |  |
| If the reviewer comments include a recommendation to cite specific previously published works, please review and evaluate these publications to determine whether they are relevant and should be cited. There is no requirement to cite these works unless the editor has indicated otherwise. | The reviewer provided comments that are not relevant to our manuscript. We have therefore not been able to work on them |
| **Reviewer #2** |  |
| The review gives a lot of background but doesn’t clearly highlight what new perspective this review adds. Right now, it feels like a collection of summaries. You should state early (in the introduction) what unique lens you’re applying compared to other reviews. Please refine and enrich your introduction by taking help from this paper and citing it. https://doi.org/10.1007/s10142-025-01613-1. https://doi.org/10.1111/cbdd.70121. https://doi.org/10.1111/cbdd.70121. doi.org/10.1007/s10989-025-10702-5. https://doi.org/10.1371/journal.pone.0317382. https://doi.org/10.1002/cpdd.1585.doi.org/10.1007/s10142-024-01425-9 Some points (gut barrier damage, microbial metabolites, dysbiosis, SCFAs) are repeated in multiple sections almost word-for-word. This makes the paper feel longer than it needs to be. These could be consolidated to make the manuscript sharper and easier to read The bacteria section is very detailed, but fungi and viruses get less critical attention. The imbalance makes those parts feel incomplete. Either expand with more critical evaluation or shorten them so expectations are aligned. Many sections describe results (“X study found this, Y study found that”), but there’s little critical discussion. For example: when probiotics show “mixed results,” the paper doesn’t explain why. A review should analyze possible reasons (differences in study design, ART status, geography, diet, etc.). The tables are useful but very text-heavy. Instead of repeating sentences, simplify to key points (“enriched,” “depleted”). This will make them faster to read. Figure 2 (gut–liver axis) is interesting but doesn’t clearly connect to HIV. Without a stronger link, it feels like a side topic. Abbreviations should be consistent (e.g., CD4⁺ vs CD4+ T cells, ART vs antiretroviral therapy). Some superscripts and subscripts are inconsistent. Standardize them. Some references are older (2014–2017). Updating with more recent studies would make the review stronger. A few sources are cited multiple times for similar points; consider diversifying references. | We sincerely thank the reviewer for taking the time to provide feedback. However, upon careful review, we noted that all the comments do not appear to relate to the content of our manuscript. It is possible that these comments were intended for a different submission. We therefore have not made changes in response to these particular comments. |
