# Supplementary material for: The experience of trial participation disclosure among sex workers in a phase IIb HIV vaccine trial: A qualitative study in urban Tanzania
Source: PLOS Glob Public Health. 2025 Nov 19;5(11):e0005511. doi: 10.1371/journal.pgph.0005511 (PMC12629454; doi:10.1371/journal.pgph.0005511)
Supplement: Attachment — Submitted filename: Comments on disclosure paper.docx [file pgph.0005511.s005.docx]

**The Experience of Trial Participation Disclosure Among Sex Workers in a Phase IIb HIV Vaccine Trial: A Qualitative Study in Urban Tanzania**

**Comments**

This is generally a well written paper and addresses an important subject. However, there are some minor issues that the authors should attend to.

1. The study title seems not to cover all the experiences described/covered in the paper. The experience of trial participation non-disclosure among sex workers, is not adequately covered in the study title.
2. On page 6, you mentioned that recordings were sent to 8 transcribers, but then on page 7, under data analysis, you say, you (authors) did the transcription to become immersed in the data. This needs to be clarified.
3. On page 8, you write, ‘Additionally, they signed a separate consent form specifically for audio recording during the qualitative interviews’. I believe the consent forms should have been signed before the qualitative interviews.
4. Long quotes from participants are usually indented on both sides and not put in quotation marks. But in your paper, they are just indented only on the left side and are put in quotation marks. Edit this appropriately.
5. On page 14, last quotation; this is the first time you write about poor adherents to the vaccine. Did the quotes that appear before this come from good adherents? The analysis and interpretation of findings should address the implications of good adherents and poor adherents in the study.
6. On page 20, edit a part of the quotation to read, ‘he believed directly that I was HIV positive’
7. On page 22, please edit this sentence for clarity, ‘Another primary reason for volunteers’ intended disclose to their participation in the HIV vaccine trial was sharing information within trusted relationships’.
8. On page 23, please edit a part of the second paragraph as follows: ‘This highlights…’
9. On page 26, in the conclusion part, the support mechanisms that should be developed to assist participants in navigating family and community dynamics that influence participation in a vaccine trial could be briefly described.
10. Did the bigger study only recruit sex workers or even other people who were not involved in sex work? This is something you can make clear in the methods section.
