# Supplementary material for: The experience of trial participation disclosure among sex workers in a phase IIb HIV vaccine trial: A qualitative study in urban Tanzania
Source: PLOS Glob Public Health. 2025 Nov 19;5(11):e0005511. doi: 10.1371/journal.pgph.0005511 (PMC12629454; doi:10.1371/journal.pgph.0005511)
Supplement: Attachment — Submitted filename: Response to Reviewers_3.docx [file pgph.0005511.s006.docx]

**The Experience of Trial Participation Disclosure Among Sex Workers in a Phase IIb HIV Vaccine Trial: A Qualitative Study in Urban Tanzania**

**Response to reviewer’s comments**

We would like to thank the reviewer for the comments provided to improve this study. We have revised the manuscript as shown in the point-to-point responses to reviewer’s comments provided in the table below.

| **Comments** | **Authors’ Response** |
| --- | --- |
| **Editor Comments** |  |
|  |  |
| **Reviewer #2** |  |
| This is generally a well written paper and addresses an important subject. However, there are some minor issues that the authors should attend to. | Thank you for the compliment |
| 1. The study title seems not to cover all the experiences described/covered in the paper. The experience of trial participation non-disclosure among sex workers, is not adequately covered in the study title. | Thank you for the comment. We had this discussion among authors before initial submission, we think the main issue is trial participation disclosure although there are other important experiences |
| 2. On page 6, you mentioned that recordings were sent to 8 transcribers, but then on page 7, under data analysis, you say, you (authors) did the transcription to become immersed in the data. This needs to be clarified. | Thank you for this observation. It was an oversight, we have made correction in the analysis since authors did not do the transcription. The statement now reads “reading the transcripts iteratively to ensure deep immersion in the data”. |
| 3. On page 8, you write, ‘Additionally, they signed a separate consent form specifically for audio recording during the qualitative interviews’. I believe the consent forms should have been signed before the qualitative interviews. | Thank you for this comment. All participants had provided written informed consent to participate in the HIV vaccine trial and its qualitative component. The initial consent covered participation in the main trial and indicated that those selected for interviews would be asked to sign an additional consent form specifically for the qualitative component, including permission for audio recording. This information is already described in the Ethics Considerations subsection. |
| 4. Long quotes from participants are usually indented on both sides and not put in quotation marks. But in your paper, they are just indented only on the left side and are put in quotation marks. Edit this appropriately. | Thank you for this comment. We have removed quotation marks from the long quotes (≥40 words) but maintained left-side indents to align with standard reporting conventions. |
| 5. On page 14, last quotation; this is the first time you write about poor adherents to the vaccine. Did the quotes that appear before this come from good adherents? The analysis and interpretation of findings should address the implications of good adherents and poor adherents in the study. | Thank you for this important observation. This was an oversight. The earlier quotes were not limited to good adherents; they represent all participants regardless of PrEP adherence. We have therefore removed this term throughout the manuscript to avoid confusion |
| 6. On page 20, edit a part of the quotation to read, ‘he believed directly that I was HIV positive’ | Thank you for this observation. We have edited the quote as suggested |
| 7. On page 22, please edit this sentence for clarity, ‘Another primary reason for volunteers’ intended disclose to their participation in the HIV vaccine trial was sharing information within trusted relationships’. | Thank you for this comment. This was a typographical error. We have corrected it to read “Another primary reason for volunteers’ intended **disclosure** to their participation in the HIV vaccine trial was sharing information within trusted relationships |
| 8. On page 23, please edit a part of the second paragraph as follows: ‘These highlights…’ | Thank you for noting this grammatical error. We have revised the sentence to read: “These highlight …” |
| 9. On page 26, in the conclusion part, the support mechanisms that should be developed to assist participants in navigating family and community dynamics that influence participation in a vaccine trial could be briefly described. | Thank you for this comment. We have added the following sentence in the conclusion “Such mechanisms could include counseling services, community sensitization activities, and structured peer-support networks to help participants manage social expectations and relational tensions arising from trial involvement.” |
| 10. Did the bigger study only recruit sex workers or even other people who were not involved in sex work? This is something you can make clear in the methods section. | Thank you for this question. The bigger PrEPVacc study recruited female sex workers only. |
