# Supplementary material for: The experience of trial participation disclosure among sex workers in a phase IIb HIV vaccine trial: A qualitative study in urban Tanzania
Source: PLOS Glob Public Health. 2025 Nov 19;5(11):e0005511. doi: 10.1371/journal.pgph.0005511 (PMC12629454; doi:10.1371/journal.pgph.0005511)
Supplement: Attachment — Submitted filename: Response to Reviewers_4.docx [file pgph.0005511.s007.docx]

**The Experience of Trial Participation Disclosure Among Sex Workers in a Phase IIb HIV Vaccine Trial: A Qualitative Study in Urban Tanzania**

**Response to reviewer’s comments**

We would like to thank the editor for the comments provided to improve this study. We have revised the manuscript as shown in the point-to-point responses to the editor’s comments provided in the table below.

| **Comments** | **Authors’ Response** |
| --- | --- |
| **Editor Comments** |  |
| Thank you for responding to the reviewers' comments. This is nearly there, but there was no revision made based on the editorial feedback. We have added this in here once more. Please can you resolve this by (a) making edits in the paper in line with this feedback (i.e. stating that a checklist like COREQ or SRQR was used), and (b) providing a supplementary document (i.e the checklist table; you will find examples of this in other qualitative publications in this journal) that can be added to the publication when it is published: | Thank you for the comments. We apologize that no revision was made based on the editorial feedback.  We have added the statement in the study design subsection that the COREQ checklist for reporting qualitative research interviews and focus groups was used in this study, including its citation. We have also uploaded the COREQ checklist as a supplementary document |
| Please also integrate a recognised check list for reporting qualitative studies. PLOS Global Public Health considers qualitative and mixed-methods studies for publication. We recommend that authors use the COREQ checklist, or other relevant checklists listed by the Equator Network, such as the SRQR, to ensure complete reporting (http://journals.plos.org/globalpublichealth/s/submission-guidelines#loc-qualitative-research). In general, we would expect qualitative studies to include the following: 1) defined objectives or research questions; 2) description of the sampling strategy, including rationale for the recruitment method, participant inclusion/exclusion criteria and the number of participants recruited; 3) detailed reporting of the data collection procedures; 4) data analysis procedures described in sufficient detail to enable replication; 5) a discussion of potential sources of bias; and 6) a discussion of limitations. | Thank you for the comment. We have integrated the COREQ checklist for reporting qualitative research interviews and focus groups to ensure complete reporting. |
